# Syncope

**Published:** 1873-10

**Authors:** E. M. Morrison

**Affiliations:** Des Moines, Iowa


### DENTAL REGISTER.

Vol. XXVII.] OCTOBER, 1873. [No. 10.

### SYNCOPE.

BY E. M. MORRISON, M. D., DES MOINES, IOWA.

Whenever there is a marked disturbance, or depression, of one or more of the great centers of vitality in any dental operation, there is cause for alarm, and most of us who have been in the profession for a long time, have sometimes felt a solicitude for the safety of our patients from causes of this character. To be master of the situation at a critical moment like this, is not always in the power of the dentist, but it is in his power, and, I may say also, clearly in the province of his duty, to be conversant with the phenomena accompanying these disturbances, and the usual means of averting or remedying them.

Syncope, which is most frequently met with by the dentist, is, according to Prof. Wood,* "a diminution or temporary ces-

* Wood's Practice of Medicine.

sation of the action of the heart, with loss of consciousness and a suspension, more or less complete, of respiration." This condition is also indicated by collapsed features, death-like paleness, and the surface of the body being cool. Prof. Taft* gives "lividness of the skin" as one of the external indications of this condition. This is a mistake : it should be pallor. Lividness is found in apnæ and cerebral congestion, but not in syncope. Both of these conditions require treatment quite different from syncope, and what would be essential in one would be detrimental in the others.

The brain, the heart and the lungs have been termed, aptly enough, the "vital tripod." As one or another of these great centers of vitality first succumbs, or gives way, the external phenomena in general are quite different, and those which are similar, have a different order of succession.

In syncope the heart is first to falter and give way, yet the brain is often primarily affected, and the syncope emotional. Papillon says :† "Very violent impressions of the feelings sometimes abruptly check the movements of the heart and produce a mortal swoon." The instances of fatality from this kind of syncope on record are very numerous, but a recital of them is not within the scope of my present purpose. Deficiency, or loss of blood is a frequent cause of this condition, but it also, according to Dr. Wood, "acts more through the brain than directly on the heart." It is further stated by the same author, that "the loss of much less blood is required to produce syncope in the erect posture than in the recumbent." This is a well known fact, yet it is a substantial proof of cerebral interposition.

The influence of anæsthetics in producing syncope is too frequently overlooked. This condition has sometimes occurred under the influence of these agents, as I have seen in my own office, when the result was fairly, as I believe, attributed to the action of nitrous oxide and chloroform.

Dr. Headland,‡ in speaking of the stages of the action of

* Taft's Operative Dentistry.

† Popular Science Monthly, July 1873.

‡ Headland on the Action of Medicine.

chloroform, says: "The inhalation should not be prolonged beyond the third stage. The pulse is still full. There are muscular movements, or even cries. In the next stage there is stertorous breathing: the eyelids no longer contract when touched with the fingers; the pulse is felt to falter. This is dangerous. To this succeed stoppage of the heart and respiration;—death."

Again, on the authority of Dr. Snow, the same author says: "A larger quantity than five per cent of the vapor in the air inspired is apt to prove suddenly fatal by causing cardiac syncope."

Papillon also says: "Anæsthetics too, like chloroform and ether, sometimes produce stronger effects than the surgeons using them desire, and occasion a state of seeming death, instead of temporary insensibility."

It has been ascertained that forty per cent. of all the accidents from anæsthetics are cases of syncope. Then, from the evidences before me, I deem it a well settled fact, that these agents are a frequent cause of syncope.

There is another point to which I wish to call attention, but it is so well expressed by Dr. Headland in the following paragraph that I cannot do better than quote him once more:

"Though chloroform rarely fails to annihilate pain, and thus greatly to diminish the terror of the patient, it will not prevent him sometimes from dying of the shock of a severe operation. An extensive injury to the body will suffice to produce prejudicial effects upon the heart and nervous system, which is independent of the feeling of pain, and may occur without moral suffering."

Then in a severe dental operation with the patient sitting in the chair, as is now the general practice, we have a formidable combination of the causes of syncope. The terror of the operation; the shock to the nervous system; the loss of blood; the erect posture; the depressing effects of the anæsthetic; and often unobserved organic lesions, are the most prominent of these causes, and they are sufficiently alarming to make the stoutest hearted shudder for the safety of his patients, and for his own reputation, if he is not ignorant of the dangers that surround him.

It is strange that more accidents have not occurred, and that the leniency of juries should continue to acquit indiscriminately in all cases. Neither of these results can be attributed to skill ; for there seems to be a total absence of this accomplishment in both instances. Dr. Kidd, of Dublin, in speaking of chloroform deaths, says : "About 90 per cent. of the cases are in trivial, small operations in persons in perfect health, viz : dentists' cases, operations of convenience." At another time the same author said : "I think I now begin to see that in hospitals and by dentists' assistants, chloroform is given too often as a matter of routine."

Syncope is distinguished from apnæ and real death, in most cases without much difficulty, and there are no other conditions very liable to be mistaken for it, or to cause confusion. In apnæ there is the same absence of pulsation, respiration and consciousness, but in syncope the countenance is pallid, and the skin pale, bloodless and cool ; while in apnæ there is lividness of the face and skin, the lips are purple, and the general signs of venous congestion are present.

By drawing the line of demarkation between organic and animal life, as did Bichat, we are enabled more readily to distinguish between apparent and real death. Vegetables have organic life in common with animals, but animals possess both combined, yet quite distinct. Animal life is first to give way, and when it is suspended, as we commonly say, without marked interruption in the organic life, there is apparent death; but even in real death the organic functions slowly subside, and in sudden deaths they give way more slowly than they do in other cases. Under favorable conditions digestion, the growth of the hair and nails, and other phenomena of organic life have been observed several hours after animal life was extinct. These phenomena depend upon the subsidiary powers. Were it not for these partial energies which outlive the principal energies, all manifestations of life would be extinct from the cessation of the heart's action, but these energies feebly support the organic functions for a time.

This tardy withdrawal of the organic forces has been the cause of much trouble and perplexity in diagnosis in the past.

and many persons have been buried alive, because their friends and even physicians could not distinguish apparent from real death. In fact, putrefaction was formerly the only test known for real death, but recent observations have thrown much light on the subject.

It has been a general belief, that the organs of the "vital tripod" could be suspended for a time at least, without death being the result, but I do not deem it probable that a full and complete cessation of any of the great centers of vitality can ever be renewed. In syncope, according to Dr. Wood: "We can generally detect the first sound of the heart, but not the second, and when observed it always gives hope of saving life." More recently and with better facilities for observation, Bauchest has proved that "in the gravest cases of fainting, the pulsations of the heart are not absolutely suspended," and that "in all the cases of seeming death, the pulsations of the heart persistently remained." It is now stated by Papillon, that 'When the heart has definitely ceased to beat, then restoration is no longer possible." This stoppage of the heart; the resistance to galvanic action; and the corpse-like rigidity which follows, are deemed sufficient to establish the fact of real death, when properly observed, and they form the basis of a correct diagnosis between this and apparent death.

In the absence of any organic disease, the cases of syncope generally recover, most of them speedily, and without much assistance, yet the loss of blood, great debility, severe shocks, the influence of anæsthetics and other causes have produced their fatal cases, when no organic disease could be detected during life, or by a *post mortem.*

The treatment of syncope consists in placing the patient in a horizontal position; administering nervous stimulants; the application of friction and warmth to the body; sprinkling the face with cold water, and other remedies of like import. The lying-down, or horizontal position is the main remedy, the *sine qua non*, without which all else is useless. The brain requires the renewal of the oxygenated blood, which the heart has failed to supply, as was proved by Brown-Sequard, to revivi fy its functions, and the head placed on a level with the heart, or below it, affords us the best means now in our hands for the accomplishment of this result. Any dentist neglecting to place his patient in a horizontal position, will fail to acquit himself of blame in case of death from syncope, and his garments will be stained with the blood of the departed. This position should be secured on the first symptoms of fainting, and may often cut short an attack.

Many of our best surgeons will not, under any circumstances, administer an anæsthetic in the erect posture, and in this respect the present practice of dentists is deemed reckless by them, and justly too, for there is no valid excuse for it, as I have before proved.* Nervous stimulants are of use occasionally, but in most cases circumstances render them unavailable. Frictions and warmth to the body are essential, as the capillaries are stimulated in this way, and without artificial warmth the body would, in most cases, speedily cool to a degree beyond restoration. Sprinkling cold water in the face may have a good effect from the shock it produces on the nervous system, and other expedients may be resorted to as the skill of the dentist may suggest, or opportunity offer.

* *Dental Register*, Sept. 1872.

